# Construction of Prognostic Risk Model of 5-Methylcytosine-Related Long Non-Coding RNAs and Evaluation of the Characteristics of Tumor-Infiltrating Immune Cells in Breast Cancer

**DOI:** 10.3389/fgene.2021.748279

**Published:** 2021-10-29

**Authors:** Zhidong Huang, Junjing Li, Jialin Chen, Debo Chen

**Affiliations:** Department of Breast Surgery, Quanzhou First Hospital of Fujian Medical University, Quanzhou, China

**Keywords:** m5C-related lncRNAs, breast cancer, risk model, nomogram, tumor-infiltrating immune cells

## Abstract

**Purpose:** The role of 5-methylcytosine-related long non-coding RNAs (m5C-lncRNAs) in breast cancer (BC) remains unclear. Here, we aimed to investigate the prognostic value, gene expression characteristics, and correlation between m5C-lncRNA risk model and tumor immune cell infiltration in BC.

**Methods:** The expression matrix of m5C-lncRNAs in BC was obtained from The Cancer Genome Atlas database, and the lncRNAs were analyzed using differential expression analysis as well as univariate and multivariate Cox regression analysis to eventually obtain BC-specific m5C-lncRNAs. A risk model was developed based on three lncRNAs using multivariate Cox regression and the prognostic value, accuracy, as well as reliability were verified. Gene set enrichment analysis (GSEA) was used to analyze the Kyoto Encyclopedia of Genes and Genomes signaling pathway enrichment of the risk model. CIBERSORT algorithm and correlation analysis were used to explore the characteristics of the BC tumor-infiltrating immune cells. Finally, reverse transcription-quantitative polymerase chain reaction was performed to detect the expression level of three lncRNA in clinical samples.

**Results:** A total of 334 differential m5C-lncRNAs were identified, and three BC-specific m5C-lncRNAs were selected, namely AP005131.2, AL121832.2, and LINC01152. Based on these three lncRNAs, a highly reliable and specific risk model was constructed, which was proven to be closely related to the prognosis of patients with BC. Therefore, a nomogram based on the risk score was built to assist clinical decisions. GSEA revealed that the risk model was significantly enriched in metabolism-related pathways and was associated with tumor immune cell infiltration based on the analysis with the CIBERSORT algorithm.

**Conclusion:** The efficient risk model based on m5C-lncRNAs associated with cancer metabolism and tumor immune cell infiltration could predict the survival prognosis of patients, and AP005131.2, AL121832.2, and LINC01152 could be novel biomarkers and therapeutic targets for BC.

## Introduction

Breast cancer (BC) is one of the leading causes of cancer-related deaths in women. According to the most recent data, in 2020, among the 19.3 million new cancer cases, female BC surpassed lung cancer for the first time by 11.7%, becoming the most commonly diagnosed cancer worldwide (Sung et al., 2021). Although the comprehensive treatment model of BC has been continuously improved in recent years, the prognosis of BC is not ideal due to recurrence and distant metastasis (Schwartz and Erban, 2017). In addition, statistics from the American Cancer Society have shown the mortality rate of breast cancer to be the highest among women aged 20–59 years (Sung et al., 2021). Early diagnosis combined with timely treatment is the key to improving the prognosis of patients with BC (DeSantis et al., 2019; Li S. et al., 2021; Lu et al., 2021); therefore, active exploration of novel prognostic biomarkers and building reliable models to further guide the diagnosis and treatment decisions of BC in future have become imperative.

In recent years, aided by the advancement of analytical chemistry tools and the rapid development of genome sequencing technology (Liu L. et al., 2020, 2021; Hasan et al., 2021a; Tang et al., 2021), epigenetic and non-coding RNA research have captured considerable attention. RNA modifications are jointly regulated by three types of effector proteins (writers, readers, and erasers) (Biswas and Rao, 2018). These form a complex protein network that regulates the gene expression process. In addition, RNA modifications, a reversible and dynamic way of regulating genetic information, play an important role in the control of genetic information, whether in coding or non-coding RNAs (Roundtree et al., 2017; Hasan et al., 2021b). Furthermore, RNA modifications have been proven to be closely related to human diseases such as cancer, metabolic diseases, vascular diseases, and neurological diseases (Jonkhout et al., 2017).

Genome-wide association studies (GWAS) have revealed that >93% of disease-linked susceptible areas are located in the non-coding region of the genome (Tak and Farnham, 2015), indicating that non-coding elements might be related to diseases. Long non-coding RNA (lncRNA), a major component of the non-coding genome (Cai et al., 2020), is a type of mRNA-like transcript with a length of >200 nucleotides (Lai et al., 2020). It plays a role in regulating gene expression at multiple levels, including epigenetics and transcriptional and post-transcriptional regulation, by interacting with other biomolecules (Wu et al., 2018). In the past decade, lncRNAs have emerged as potentially important regulators of pathological processes, including cancer. For example, lncRNA *XIST* sponges miR-34a suppresses the expression of WNT1 by binding to its 3′ untranslated regions (3′-UTR), thus inhibiting the proliferation of colon cancer (Sun et al., 2018). The lncRNA CTBP1-AS represses CTBP1 expression *via* a persisting-cell-stimulating factor (PSF)-dependent mechanism that affects prostate cancer progression (Takayama et al., 2013).

**FIGURE 1:**
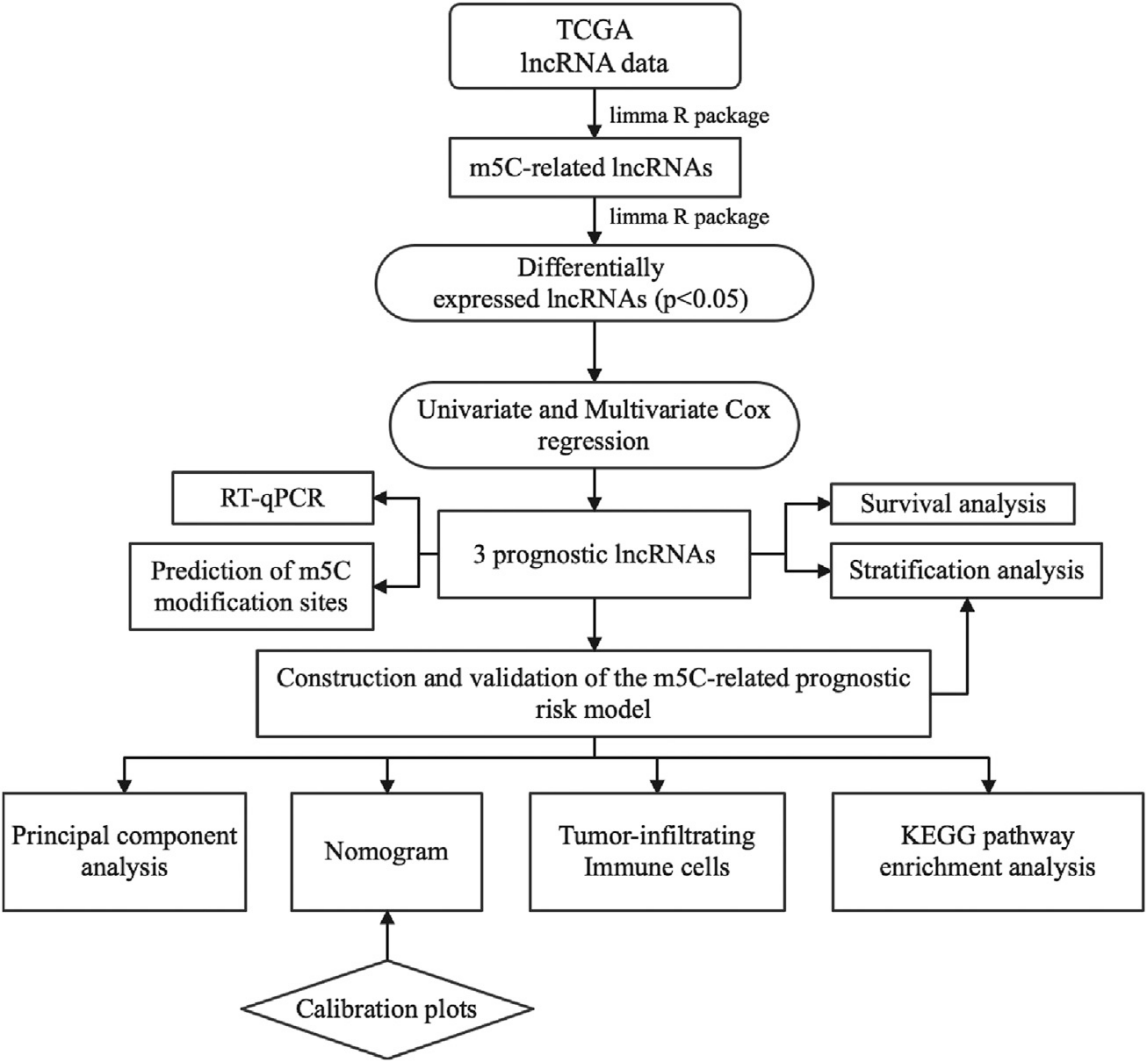
Flowchart for establishing and evaluating prognostic risk model.

RNA modification-related lncRNAs have been confirmed to be associated with head and neck squamous cell carcinoma, colorectal cancer, hepatocellular carcinoma, and liver cancer (Tu et al., 2020; Xue et al., 2020; Zuo et al., 2020; Feng et al., 2021). However, the role of 5-methylcytosine-related lncRNAs (m5C-lncRNAs) in BC cells is still poorly defined. The current study aimed to screen differentially expressed and prognostic m5C-lncRNAs to build a risk model that can reliably predict the prognosis of patients. This could aid the built of a nomogram, which might help with clinical decisions. Lastly, we explored the potential signaling pathways of m5C-lncRNA-mediated tumor development through gene set enrichment analysis (GSEA) and the correlation between risk model and tumor-infiltrating immune cells (TIICs) (Figure 1 **)**.

## Materials and Methods

### Data Collection and Identification of m5C-lncRNAs

RNA-sequencing, patient characteristics, and clinical information of BC (1109 samples) and normal breast tissue (113 samples) were obtained from The Cancer Genome Atlas (TCGA) (https://portal.gdc.cancer.gov/) database (Table 1). Genome names and genotypes were annotated using the Genome Reference Consortium Human Build 38 (GRCH38) data from the Ensembl database (https://asia.ensembl.org) to further screen for lncRNAs and mRNAs. The following 15 m5C regulatory factors were collected based on previous reports: NSUN1, NSUN2, NSUN3, NSUN4, NSUN5, NSUN6, NSUN7, DNMT1, DNMT2, DNMT3A, DNMT3B, TET2, ALYREF, TRDMT1, and YBX1. A total of 422 m5C-lncRNAs were screened using the limma package from R.

**TABLE 1:** Clinical characteristics of patients with breast cancer.

Variables	No. of patients	Percentage (%)
**Age (years)**		
≤55	471	42.9
>55	626	57.1
Unknown	19	1.7
**Gender**		
Female	1085	98.9
Male	12	1.1
**Pathological stage**		
I	183	16.7
II	621	56.6
III	249	22.7
IV	20	1.8
Unknown	24	2.2
**T stage**		
T1	281	25.6
T2	635	57.9
T3	138	12.6
T4	40	3.6
Unknown	3	0.3
**N stage**		
N0	516	47.0
N1	364	33.2
N2	120	10.9
N3	77	7.0
Unknown	20	1.8
**M stage**		
M0	912	83.1
M1	22	2.0
Unknown	163	14.9

### Screening and Validation of BC-Specific m5C-lncRNAs

We used a two-step screening method to identify the candidate BC-specific lncRNAs. First, differential m5C-lncRNA expression analysis between BC and normal breast tissue was performed using limma package, and 334 differentially expressed lncRNAs were screened out (*p* < 0.05). Five prognosis-related lncRNAs were identified using univariate Cox regression analysis, and the significant lncRNAs were subsequently included in a multivariate Cox regression analysis (*p* < 0.05). Three m5C-lncRNAs were selected. To further verify the tissue specificity of lncRNAs in BC, Kaplan–Meier (KM) survival analysis and subgroup analysis for the clinicopathological characteristics of the three lncRNAs were performed.

### Construction of Prognostic Model and Nomogram

Based on BC-specific m5C-lncRNAs, we constructed a prognostic risk model of m5C-lncRNAs in patients with BC. Multivariate Cox regression was used to calculate their coefficients (Coefi). Then, the fragments per kb per million mapped reads value (*xi*) of each m5C-related lncRNA and Coefi were used to develop a formula for the risk score:

$$Risk\;score\;=\;\sum_{i=1}^{n}coefi\;\cdot \;xi$$

All patients with BC were divided into two subgroups (low- and high-risk groups) using the median of all patient risk scores as the cut-off. Stratified analysis of clinical characteristics and difference in expression of the three prognostic lncRNAs in the different risk groups were analyzed by creating a heat map. To test the prognostic value of the risk model, the correlation between patient survival and risk score was determined. Univariate and multivariate Cox analyses, receiver operating characteristic (ROC) curve, as well as principal component analysis (PCA) further proved the independent prognostic value, sensitivity, specificity, and reliability of the risk model. To enable the risk model to make clinical diagnosis and treatment decisions, we constructed a nomogram based on independent prognostic factors, and calibrated the same to evaluate the predicted probabilities of the nomogram.

### KEGG Pathway Enrichment Analysis

We explored the potential signaling pathways implicated in the m5C-lncRNA risk model using KEGG pathway enrichment analysis and the GSEA software. The reference gene set was retrieved from c2.cp.kegg.v7.4. symbol files; pathways with | NES | >1; NOM *p*-value < 0.05, and FDR q-value <0.25 were defined as significantly enriched.

### Correlation Analysis of TIICs

Using CIBERSORT L22 as the reference (Newman et al., 2015), we analyzed the m5C-lncRNAs expression matrix using CIBERSORT R script acquired from the CIBERSORT website (http://cibersort.stanford.edu/), and the relative proportions of 22 immune cells were calculated. For each tumor sample, the sum of relative proportion scores of all immune cells was 1. The immune cell subtypes with lower scores were removed. The Spearman correlation analysis was performed on the remaining 21 immune cell types. We identified the low- and high-risk groups to screen the differentially infiltrated immune cells and investigated whether there was a difference in infiltration across the different groups.

### RNA Isolation and Quantitative Real Time Polymerase Chain Reaction

To further verify the differential expression of three m5C-lncRNAs in BC, we extracted RNA from fresh frozen tissues using TRIzol reagent (Invitrogen, Carlsbad, CA, United States) and tested the expression levels of all three lncRNAs. cDNA was synthesized using a reverse transcription kit (TaKaRa, Japan). The LightCycler 480 Real-Time PCR System was used to detect the relative expression of the target lncRNAs. GAPDH was used as an internal normalization control. We collected 31 pairs of fresh BC and paracarcinoma tissues from the Department of Breast Surgery, Quanzhou First Hospital, between 2020 and 2021 (Table 2). The primer sequences are listed in Supplementary Table S1.

**TABLE 2:** Clinical information of 31 patients with breast cancer in the qPCR cohort.

Variables	No. of patients	Percentage (%)
**Age (years)**		
≤55	4	12.9
>55	27	87.1
**Gender**		
Female	31	100.0
Male	0	0.0
**Pathological stage**		
I	6	19.4
II	15	48.4
III	10	32.3
IV	0	0.0
**T stage**		
T1	11	35.5
T2	18	58.1
T3	2	6.5
T4	0	0.0
**N stage**		
N0	13	41.9
N1	8	25.8
N2	4	12.9
N3	6	19.4
**M stage**		
M0	31	100.0
M1	0	0.0

### Prediction of m5C Sites on the Three lncRNAs

RNAm5Cfinder (http://www.rnanut.net/rnam5cfinder/); (Li J. et al., 2018), iRNAm5C-PseDNC (http://www.jci-bioinfo.cn/iRNAm5C-PseDNC); (Qiu et al., 2017), and iRNA-m5C (http://lin-group.cn/server/iRNA-m5C/service.html); (Lv et al., 2020) databases were used to further verify the m5C modification sites on lncRNAs.

### Statistical Analysis

Statistical significance was set at *p* < 0.05, and Kruskal-Wallis or Wilcoxon test was used for comparing the differential expression of m5C-lncRNA between different groups. The KM and life table method was used to estimate survival, and log-rank test was employed to compare the survival curves across the different groups. Pearson’s correlation coefficient was used to determine the correlation between lncRNAs and infiltration of tumor immune cells.

## Results

### Identification of BC-specific m5C-lncRNAs

**FIGURE 2:**
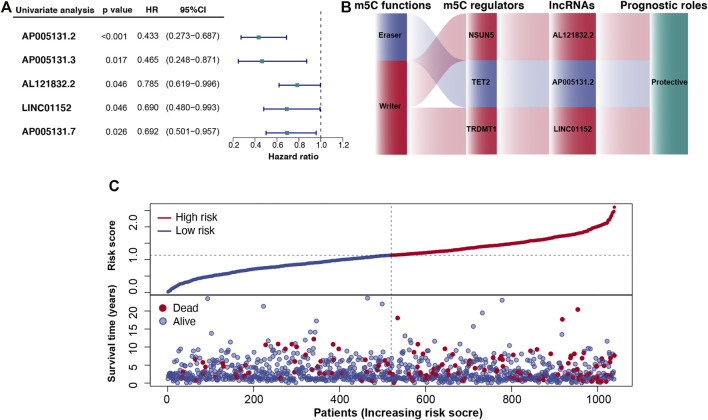
Cancer-specific m5C-lncRNAs and their co-expression networks with m5C regulators in breast cancer (BC). **(A)** Forest diagram of univariate Cox regression analysis of m5C-lncRNAs. **(B)** Co-expression networks of the three m5C-lncRNAs screened using multivariate Cox regression analysis. **(C)** Distributions of risk scores and survival status of patients with BC based on m5C-lncRNA signature from the TCGA dataset.

We used differential expression and survival analyses to select BC-specific lncRNAs from 422 m5C-lncRNAs deposited in the TCGA dataset. A total of 334 differentially expressed lncRNAs between BC and normal breast tissues were screened (*p* < 0.05). Univariate Cox regression analysis was performed to analyze the prognostic factors of m5C-lncRNAs based on these lncRNAs (*p* < 0.05) (Figure 2A).

### Construction of the m5C-lncRNA-Related Risk Model

The prognostic lncRNAs identified following univariate Cox regression analysis were included in the multivariate analysis. Three independent prognostic lncRNAs (AP005131.2, AL121832.2, and LINC01152) (Supplementary Table S2) were screened to build a three-gene risk model. Coefficients of m5C-lncRNAs from the multivariate Cox regression were used as coefficients of corresponding factors in the risk model (Supplementary Table S3). As shown in Figure 2B, the co-expression network that combined the function of m5C, m5C regulators, selected m5C-lncRNAs, and their prognostic roles indicated NSUN5, TET2, and TRDMT1 modified three lncRNAs to be protective factors.

According to the risk score, patients with BC were divided into high- and low-risk groups. Figure 2C shows the relationship between risk score and the corresponding survival status, suggesting that higher the risk scores, higher the number of deaths.

### Correlation Between Risk Model, Three m5C-lncRNAs, and Clinical Variables

**FIGURE 3:**
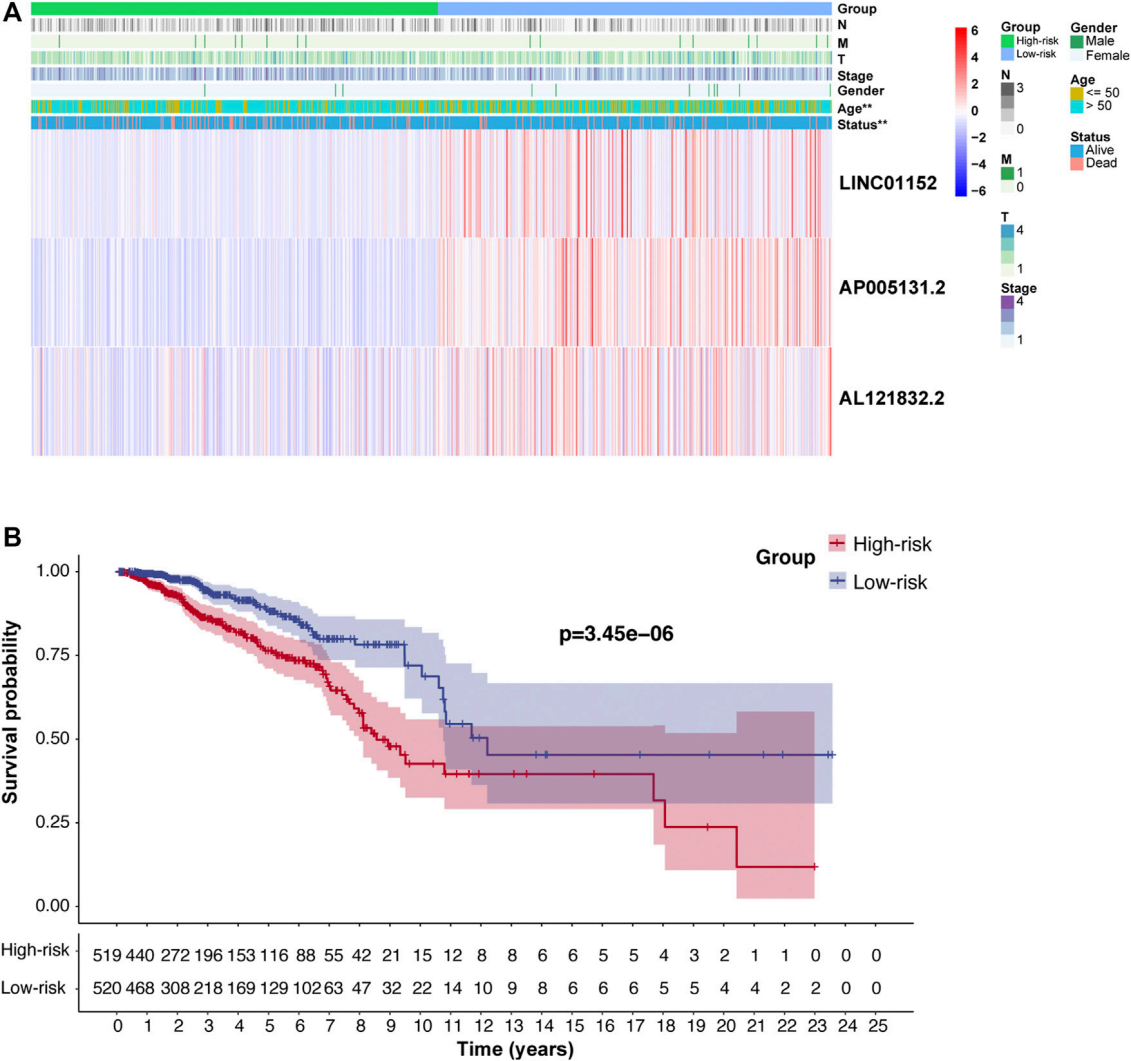
The clinicopathological features and prognosis of patients in the high- and low-risk groups. **(A)** Heatmap showing the clinicopathological characteristics and expression levels of the three m5C-lncRNAs in the high- and low-risk groups. **(B)** Prognostic value of the model based on Kaplan–Meier survival analysis. (**p < 0.01).

**FIGURE 4:**
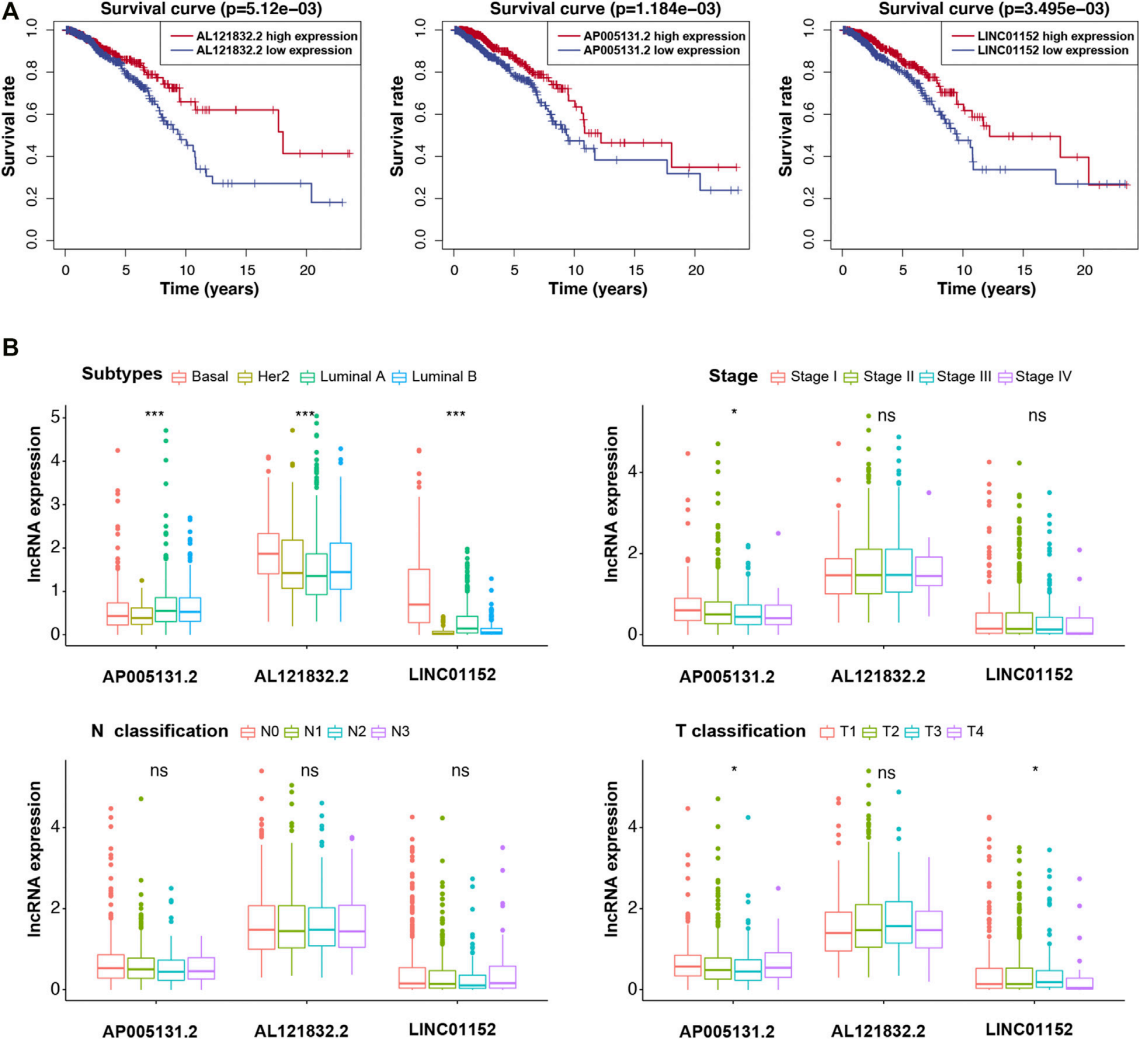
Survival analysis and clinicopathological characteristics of the three m5C-lncRNA in high- and low-expression groups. **(A)** Kaplan–Meier survival curve of high- and low-expression groups. **(B)** Analysis of clinicopathological features. (*p < 0.5, ***p < 0.001).

We further analyzed the relationship between the risk model, m5C-lncRNAs, and different clinicopathological features. The age (*p* < 0.01) and survival status (*p* < 0.01) were significantly different between the high- and low-risk groups. Moreover, the three prognostic m5C-lncRNAs were differentially expressed in the high- and low-risk subgroups; high expression of AP005131.2, AL121832.2, and LINC01152 was associated with low-risk scores. However, there was no significant difference between the pathological stage and TNM stage (Figure 3A). The risk model prognostic value was assessed using survival analysis. Results indicated that the high-risk group was associated with worse outcomes compared to those observed in the low-risk group (*p* < 0.05) (Figure 3B). KM survival analysis showed a close relationship of the three lncRNAs with the overall survival (OS) of BC patients (Figure 4A). To further explore the value of risk model in BC, subgroup analysis of the three prognostic lncRNAs was performed based on molecular subtypes, histology stage, T stage, and N stage. As shown in Figure 4B, all lncRNAs showed a significant correlation with BC molecular subtypes (*p* < 0.001). In terms of histological stage, the expression of AP005131.2 in the early stage was significantly higher than that in the later stage (*p* < 0.05). For T stage, AP005131.2 and LINC01152 were differentially expressed in each group (*p* < 0.05). However, expression of AP005131.2, AL121832.2, and LINC01152 was not significantly different across groups in the N stage.

### Validation of the m5C-lncRNA Risk Model

**FIGURE 5:**
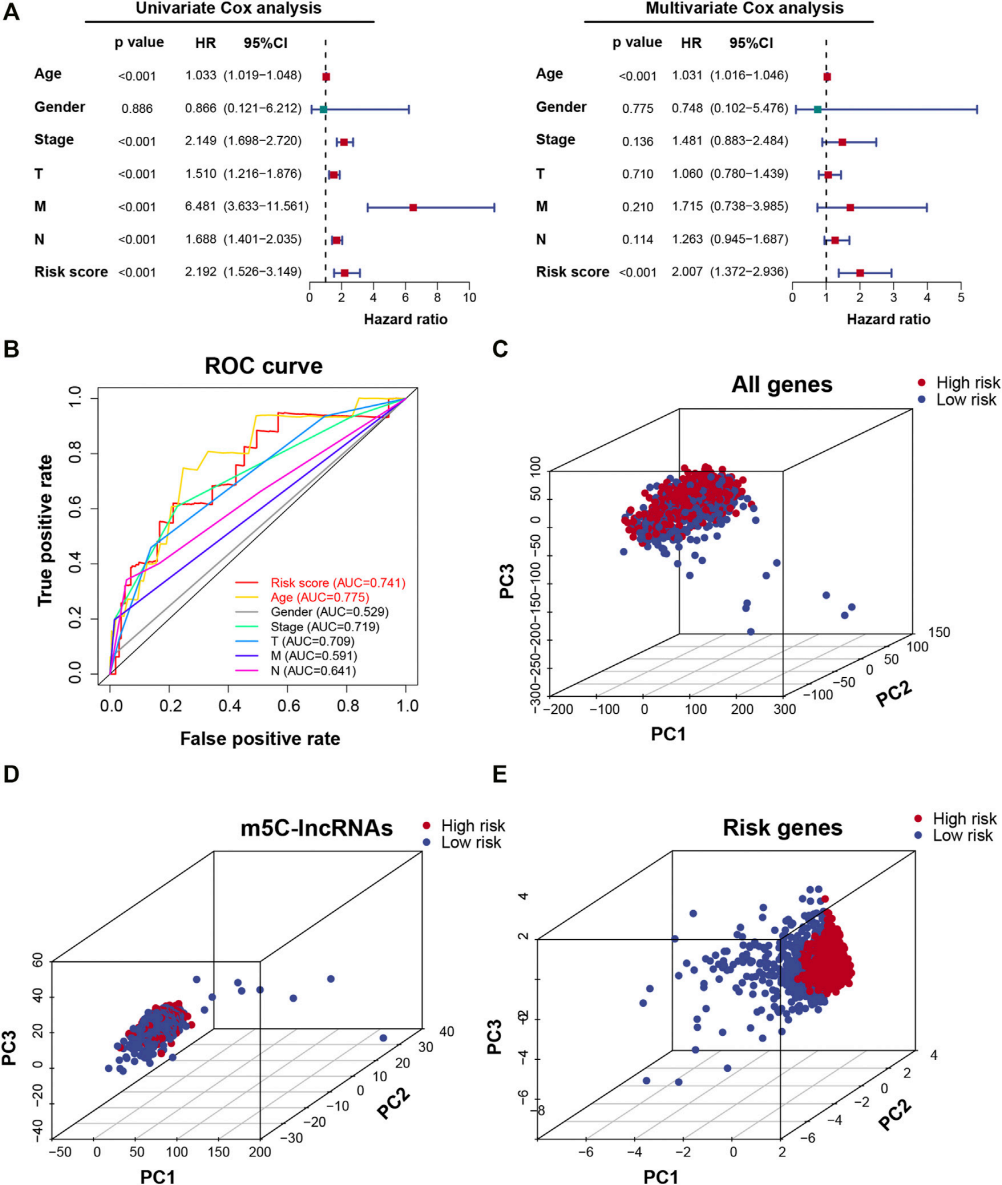
Prognostic risk model verification. **(A)** Univariate and multivariate Cox regression analyses of risk scores combined with clinicopathological characteristics. **(B)** The receiver operating characteristic (ROC) curve verifies the sensitivity and reliability of the risk model. **(C–E)** Principal component analysis between low- and high-risk groups based on the signature of all genes, m5C-lncRNAs, and risk genes.

Univariate and multivariate Cox regression analyses were used to analyze the independent prognostic value of the risk model and clinical variables. The univariate and multivariate analyses revealed that age (continuous variable; HR = 1.033 and 1.031, respectively, 95% CI: 1.019–1.048 and 1.016–1.046, respectively; *p* < 0.001) and risk score (continuous variable; HR = 2.192 and 2.007, respectively, 95% CI: 1.526–3.149 and 1.372–2.936, respectively; *p* < 0.001) can be independent of gender, pathological stage, and TNM stage while being important prognostic factors (Figure 5A) Time-dependent ROC curves were used to evaluate the prognostic and predictive ability of age and risk score. Results revealed that, compared to other predictors, age and risk score had higher precision (AUC = 0.775, 0.741, respectively) (Figure 5B). PCA was used to show the different distribution patterns of m5C between the two risk groups across all genes, m5C-lncRNAs, as well as the risk gene expression profiles and found that patients in the risk gene group were significantly spread in different directions, showing the sensitivity, specificity, and positive and negative predictive values of the risk model (Figures 5C–E). The above results indicated that the m5C-lncRNA risk model is a significant independent prognostic indicator, applicable for clinical prognosis evaluation in patients with BC.

### Construction of m5C-lncRNA Nomogram Based on the Risk Model

**FIGURE 6:**
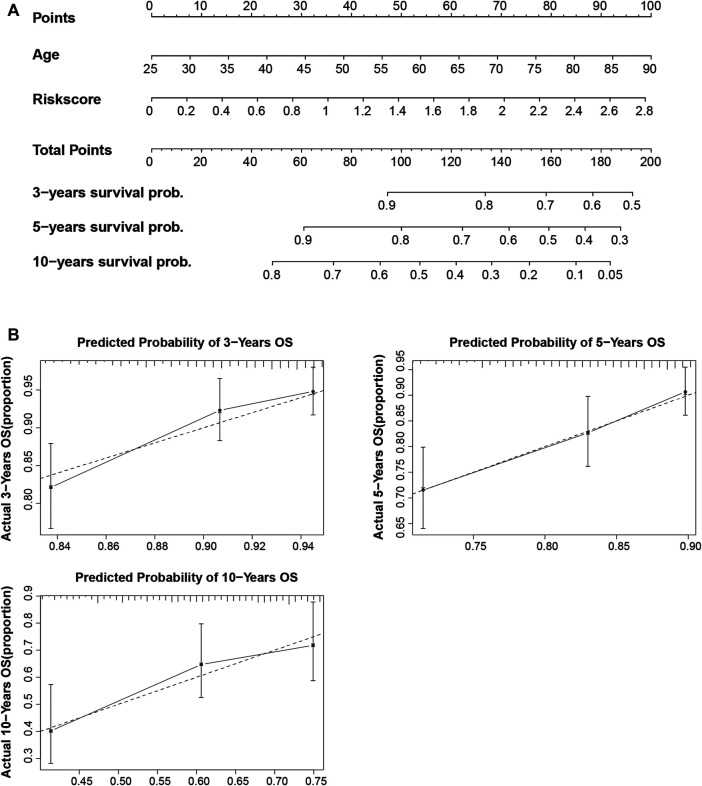
Construction and calibration of the nomogram. **(A)** Construction of a nomogram based on age and risk score as independent prognostic factors. **(B)** The calibration curve revealed that the nomogram was well calibrated; the 3-, 5-, and 10-years overall survival showed an optimal agreement between the actual observation and nomogram prediction.

To establish a clinically available quantitative tool for predicting patient prognosis, we constructed a nomogram using the risk score and age (Figure 6A). Calibration plots showed that the observed *vs*. predicted rates of 3-, 5-, and 10-years overall survival (OS) were in perfect concordance (Figure 6B).

### Verification of *in vitro* Expression Level and Prediction of m5C Modification Sites on Three m5C-lncRNAs

We identified AP005131.2, AL121832.2, and LINC01152 as the lncRNAs with the highest prognostic value. We further verified the expression of the m5C-lncRNAs in BC and paracancerous tissues. The expression levels of AP005131.2 and AL121832.2 were significantly lower in tumor tissues than in paracancerous tissues (*p* < 0.05). While there was no statistically significant difference in the expression of LINC01152, we observed a trend towards lower expression levels in tumor tissues (Supplementary Figure S1). The results were consistent with the TCGA cohort. We further investigated the m5C modification sites on three m5C-lncRNAs using three online databases, which are based on different algorithms (RNAm5Cfinder, iRNAm5C-PseDNC, and iRNAm5C) (Supplementary Table S4); the result confirmed that the three m5C-lncRNAs were lncRNA modified by m5C.

### GSEA and the Correlation of TIICs

**FIGURE 7:**
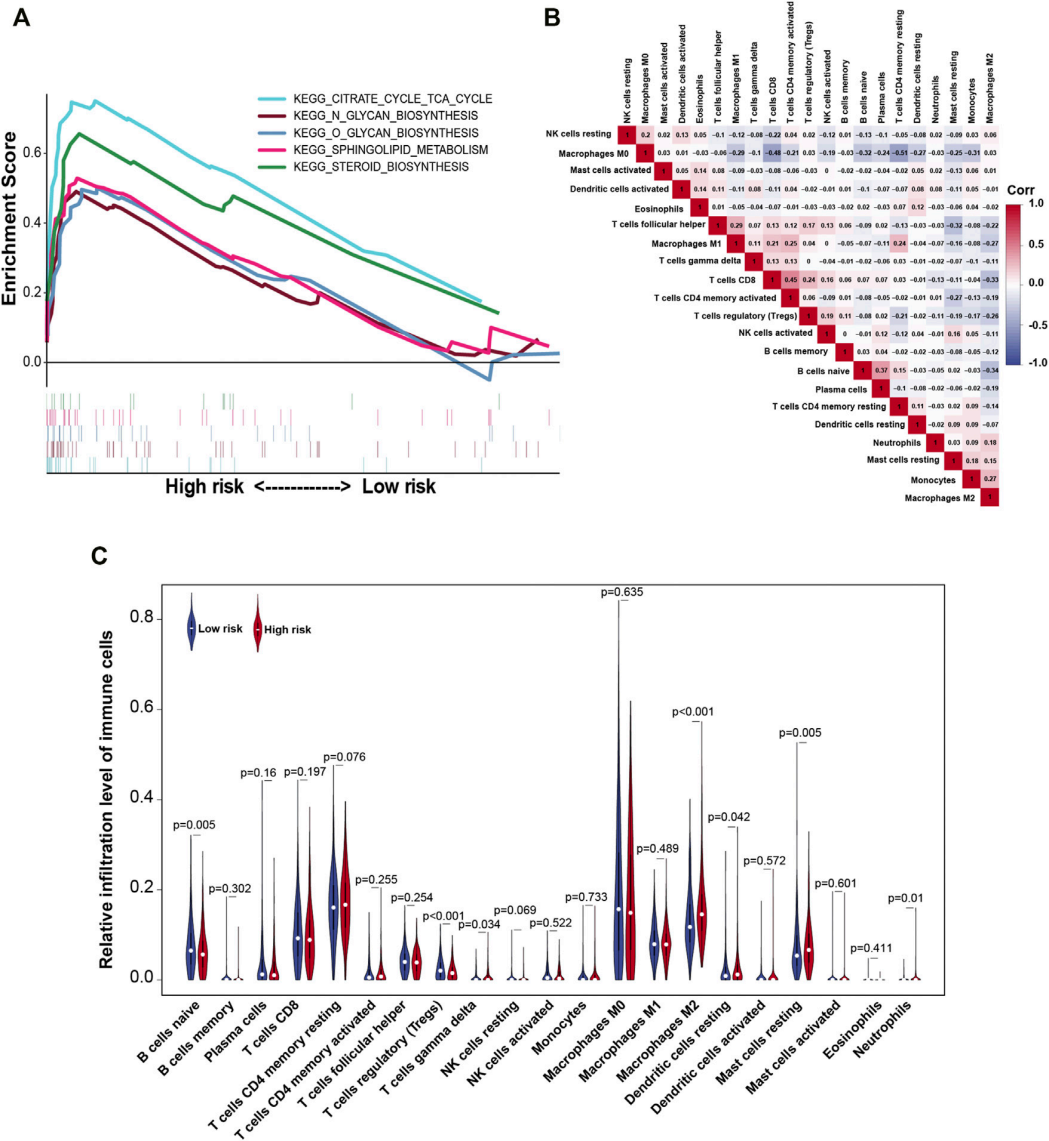
Gene set enrichment analysis (GSEA) and the correlation between risk model and tumor-infiltrating immune cells. **(A)** Enrichment analysis of gene signaling pathways in risk models. **(B)** Correlation between the expressions of tumor-infiltrating immune cells in BC. **(C)** Violin plot of relative infiltration level of tumor immune cells in the high- and low-risk groups. Corr, Spearman correlation coefficient.

The alteration of risk model associated multiple cancer signaling pathways were identified through GSEA. The analysis showed that the high-risk group was mainly enriched in the following terms: citrate cycle (TCA cycle), glycan biosynthesis, sphingolipid metabolism, and steroid biosynthesis (Figure 7A). Based on the important role of TIICs in tumor occurrence and development, we analyzed the heterogeneity of TIICs in BC based on the risk score. First, we analyzed the correlation between 21 types of immune cells. Activated memory CD4 T cells had a strong positive correlation with CD8 T cells (r = 0.45), followed by the plasma cells and naive B cells (r = 0.37). Conversely, resting CD4 memory T cells had a strong negative correlation with macrophages M0 (r = −0.51) in BC tissues (Figure 7B). Next, we analyzed the expression levels of the 21 types of immune cells between high- and low-risk groups. The proportion of naive B cells and regulatory T cells in the low-risk group was higher than that in the high-risk group (*p* < 0.01). Additionally, the proportion of gamma delta T cells, macrophages M2, resting dendritic cells, resting mast cells, and neutrophils in the high-risk group was higher than that in the low-risk group (*p* < 0.05) (Figure 7C). These findings indicated that the risk model can distinguish the characteristics of TIICs in BC tissues.

## Discussion

BC is one of the most frequent malignancies with the highest mortality rate worldwide (DeSantis et al., 2019). Although a variety of therapeutic options (surgery, chemotherapy, radiotherapy, and immunotherapy, etc.) have made significant progress in recent years (Zhou et al., 2012; Pan et al., 2021), the prognosis of BC requires further improvement. Precise diagnosis and treatment are essential for improving the survival of patients with BC. Thus, comprehensive research would be required to explore the effective therapeutic targets and tailor precise treatment plans for each patient. Currently, more than 150 RNA post-transcriptional modification methods are in use, of which m6A, m5C, and m1A are the most common modifications (Zhao et al., 2017) involved in cell proliferation regulation and disease progression. Previous studies have found that RNA post-transcriptional modification play an important role in lncRNA (Liu H. et al., 2020; Yi et al., 2020); however, the m5C modification of lncRNA is still poorly understood. In the present study, we screened m5C-lncRNAs for prognostic value as well as abnormal expression, and using multivariate Cox and risk scoring methods, we constructed a prognostic prediction model and combined the same with clinicopathological features and tumor immune cell infiltration to explore the role of m5C-lncRNAs in BC.

LncRNAs are transcripts of more than 200 nucleotides (Ma et al., 2016) and play an important role in the multi-stage (epigenetics, transcription level, post-transcriptional level) occurrence and development of various malignancies (Chalei et al., 2014; Gong et al., 2015; Qin et al., 2016; Goyal et al., 2017) including mediating the interaction between DNA and protein, adsorbing miRNA, and regulating the expression of target proteins. Increasing evidence indicates that the expression level of lncRNA changes under pathological conditions, thus affecting cancer progression. For example, m6A methyltransferase-like 3 has been reported to stabilize the expression of LINC00958, which acts as a competitive endogenous RNA of *miR-378a-3p* to upregulate the expression of YY1; thereby facilitating BC progression (Rong et al., 2021). Studies have revealed that there are m6A modification sites on lncRNA NEAT1, which is related to bone metastasis in prostate cancer. The lncRNA NEAT1 acts as a bridge between CYCLINL1 and CDK19 to promote Pol II ser2 phosphorylation, which might represent a new target for the treatment and diagnosis of bone cancer metastasis (Wen S. et al., 2020). However, currently there are only few studies reporting the effects of m5C-lncRNAs. In hepatocellular carcinoma (HCC), m5C-related RNA methyltransferase *NSUN2* targets lncRNA *H19* and stabilizes its expression. LncRNA *H19* further binds to the known oncoprotein G3BP1, leading to downstream MYC accumulation, subsequently promoting the proliferation of HCC cells (Sun et al., 2020). In esophageal squamous cell carcinoma (ESCC), lncRNA *NMR* methylated by *NSUN2* combines with the chromatin regulator BPTF to promote the expression of MMP3 and MMP10 *via* the ERK1/2 pathway; thus, promoting the progression of ESCC (Li Y. et al., 2018). However, the regulatory role of m5C on lncRNAs in BC has not been reported yet.

In this study, we used BC-specific m5C-lncRNAs AP005131.2, AL121832.2, and LINC01152 to construct a risk model. Univariate Cox, multivariate Cox, and ROC curve analyses confirmed that the risk model can be independent of the traditional TNM stages and other clinical features and has good prognostic and predictive values. This is the first model based on m5C-related lncRNAs that is predictive of BC prognosis, offering new directions for research and clinical management of this common cancer. Besides, compared with other similar RNA methylation-related risk models, this model also has a high degree of prediction sensitivity and specificity (Zhang et al., 2020; Wang et al., 2021; Zhang et al., 2021). To apply the prognostic model in clinical practice, we built a nomogram based on age, stage, and risk score, which can easily predict the prognosis of patients over 1, 5, and 10 years. Furthermore, PCA showed that risk gene can better illustrate the m5C characteristics. The results presented above indicated that AP005131.2, AL121832.2, and LINC01152 can be used as potential biomarkers, and a reliable risk model and risk-model-based nomograms are critical for providing the necessary evidence for clinical adoption and for driving continual improvement in patients with BC.

Currently, only LINC01152 has been reported among the three m5C-lncRNAs (Chen et al., 2019, 23; Wu et al., 2021). In glioblastoma multiforme, LINC01152 can upregulate the expression of MAML2 *via* the Notch signaling pathway to promote glioblastoma multiforme tumorigenesis. It can bind to the promoter region of IL-23, promote its transcriptional activity, and upregulate the levels of Stat3 and p-Stat3, resulting in the subsequent progression of HCC. Combined with bioinformatics analysis and tissue level verification, AP005131.2, AL121832.2, and LINC01152 generally showed low expression, which was related to better prognostic value in BC. BC is a highly heterogeneous tumor based on gene expression profiles, and can be divided into three subtypes: luminal (ER-and/or PR-positive), HER2 enriched (HER2 positive), and basal subtypes (ER, PR, and HER2 negative) (Yeo and Guan, 2017). Different subtypes have different pathological processes. Most of the autophagy-related lncRNAs had been previously reported to be significantly related to molecular subtypes of BC, indicating that autophagy-related lncRNAs may participate in the regulation of ER, PR, and HER2 status (Li X. et al., 2021). In this study, the expression of three m5C-lncRNAs was significantly correlated with different molecular subtypes, indicating that m5C-lncRNAs might participate in the regulation of ER, PR, and HER2 status.

We explored the biological processes that m5C-lncRNAs may participate in. GSEA demonstrated that in patients with BC and high-risk scores, cell signaling pathways were mainly enriched in the citrate cycle (TCA cycle), glycan biosynthesis, sphingolipid metabolism, and steroid biosynthesis. We found that the risk models were mainly enriched in metabolic-related pathways. Studies have shown that cholesterol-derived metabolites play a critical functional role in supporting cancer progression and suppressing immune responses (Huang et al., 2020). In BC, sphingosine kinase 1, which is necessary for the generation of S1P and its receptor S1PR1 can induce the release of proinflammatory cytokines, macrophage infiltration, and tumor progression (Nagahashi et al., 2018). The specific mechanism by which m5C-related lncRNAs participate in the regulation of metabolic pathways requires further *in vivo* and *in vitro* experimentation.

In recent years, research on the effects of lncRNAs in TIM has received widespread attention. Immune-related lncRNAs have important prognostic value in various cancers, such as lung adenocarcinoma, HCC, and low-grade glioma (Wen J. et al., 2020; Hong et al., 2020; Li et al., 2020). Recently, a prognostic model has been built based on four immune-related lncRNAs that can better predict the prognosis of patients with BC (Shen et al., 2020). Here, we provided an in-depth analysis of the relationship between the risk model and TIM cells. Our findings highlighted that the high- and low-risk groups significantly distinguished the characteristics of naive B cells, regulatory T cells, gamma delta T cells, macrophages M2, resting dendritic cells, resting mast cells, and neutrophils, among which naive B cells and regulatory T cells exhibited a higher degree of infiltration in the low-risk group than in the high-risk group. Alternatively, gamma delta T cells, macrophages M2, resting dendritic cells, resting mast cells, and neutrophils exhibited a higher degree of infiltration in the high-risk group than in the low-risk group. Altogether, the risk model could evaluate the tumor infiltration of immune cells to analyze the tumor immune characteristics, thereby determining the prognosis of patients with BC.

However, there are some limitations associated with our research. First, we screened m5C-related lncRNAs using the TCGA database and constructed a risk model based on the same database. Secondly, owing to the limitations of an imperfect annotation of lncRNAs and a dearth of complete lncRNA-seq data in cancer, the risk model could not be further verified. In addition, we examined the expression levels of the three m5C-lncRNAs at histological level but did not perform further *in vivo* and *in vitro* analyses. We did not perform further experiments, such as MeRIP-seq and RNA-BisSeq, to verify the m5C modification sites on lncRNAs. Further studies are warranted to validate our findings in future.

In conclusion, the risk model constructed based on the three m5C-lncRNAs (AP005131.2, AL121832.2, and LINC01152) has independent prognostic value and extremely high reliability in BC; thus, providing clues for in-depth research on m5C modification sites in lncRNAs. Moreover, the risk model for BC was translated into a nomogram, providing a convenient and quantitative prognostic prediction tool for clinicians, new insights for understanding immune cell-specific genes in tumor regulation, and possibly improving the ability to individualize treatment for patients with BC.

## Data Availability

The original contributions presented in the study are included in the article/Supplementary Material, further inquiries can be directed to the corresponding author.
