# Supplementary material for: Construction of Prognostic Risk Model of 5-Methylcytosine-Related Long Non-Coding RNAs and Evaluation of the Characteristics of Tumor-Infiltrating Immune Cells in Breast Cancer
Source: Front Genet. 2021 Oct 29;12:748279. doi: 10.3389/fgene.2021.748279 (PMC8585929; doi:10.3389/fgene.2021.748279)
Supplement: Supplementary file 1 [file DataSheet1.docx]

Supplementary Material

| Primer | 5' to 3' |
| --- | --- |
| AP005131.2-F | AGAAAGGCAAGGTTTTGCTGAA |
| AP005131.2-R | TTTTAGAAAGGAGAAGGAACCA |
| AL121832.2-F | ATCTGAAACACCCGACCACTCT |
| AL121832.2-R | GGTCCAAGGGTATGCACCTCTA |
| LINC01152-F | TGGGGACAGATTTAACCCAAAG |
| LINC01152-R | CCAAGTTCGTGCCATTGTATTC |
| GAPDH-F | GGTGTGAACCATGAGAAGTATGA |
| GAPDH-R | GAGTCCTTCCACGATACCAAAG |

**Supplementary Table 1.** The primer sequences involved in this study.

| lncRNA | HR | HR.95L | HR.95H | pvalue |
| --- | --- | --- | --- | --- |
| AP005131.2 | 0.41929285 | 0.26497383 | 0.66348626 | 0.00020564 |
| AL121832.2 | 0.79780893 | 0.63208142 | 1.00698908 | 0.04725752 |
| LINC01152 | 0.66292022 | 0.45733663 | 0.96091848 | 0.02997367 |

**Supplementary Table 2.** Multivariate Cox regression analysis identified m5C-related lncRNAs with important prognostic value.

| lncRNA | Coefficient |
| --- | --- |
| AP005131.2 | -0.8691857 |
| AL121832.2 | -0.2258861 |
| LINC01152 | -0.4111006 |

**Supplementary Table 3.** Coefficients of three m5C-lncRNAs in risk model.

|  | RNAm5Cfinder | iRNAm5C-PseDNC | iRNAm5C |
| --- | --- | --- | --- |
| AP005131.2 | 8 | 0 | 49 |
| AL121832.2 | 36 | 0 | 51 |
| LINC01152 | 19 | 3 | 87 |

**Supplementary Table 4.** Number of m5C Modification Sites on three lncRNAs predicted from RNAm5Cfinder, iRNAm5C-PseDNC, iRNAm5C online databases.


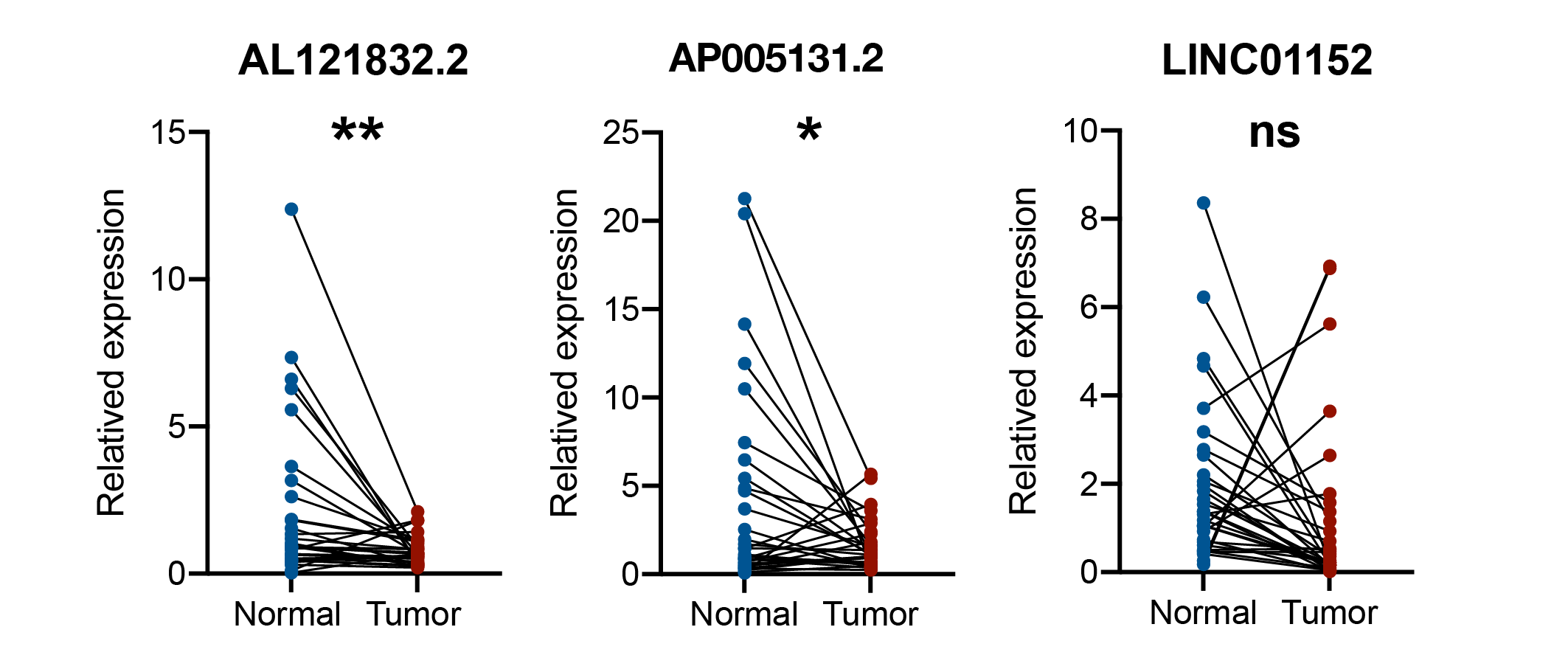


Supplementary Figure 1. RT-qPCR verified the expression levels of three m5C-lncRNAs. (*p < 0.5, **p < 0.01)
